# Reply to Drs. Capucci, Lavazza, and Mead

**DOI:** 10.3201/eid0402.980238

**Published:** 1998

**Authors:** Alvin W. Smith, Neil J. Cherry, David O. Matson

**Affiliations:** * Oregon State University, Corvallis, Oregon, USA;; † Lincoln University, Christchurch, New Zealand;; ‡ Center for Pediatric Research, Norfolk, Virginia, USA


The RHD exposure categories of "low" and "high" used by Mead et al. and mentioned in the first column of page 18 (1) are not related to the categories of "low" and "high" given in the same paragraph at the top of the second column. The reader might easily assume that it was Mead et al. who considered that Jul–Dec 1995 was "a low exposure period." This is not so—such a classification is made by Smith et al.

Further, the reader might assume that it was the study by Mead et al. that concluded "that exposure to RHD virus remains a plausible explanation for increased disease incidence." Again this is an inference drawn by Smith et al. and is the opposite of the conclusion of Mead et al.

The basis of exposure in the study by Mead et al. is at an individual level—the respondents were chosen either because they had been handling rabbits or as controls in determining the level of disease. In contrast, Smith et al. consider exposure at a broad environmental level and disregard whether the respondents had been handling infected rabbits or not. Actually, more contact with rabbits occurred during the first half of the study than during the second.

Smith et al. do not mention the conclusions of Mead et al.: These neither showed any significant difference between levels or types of illness in those exposed and those not exposed to RHD virus nor demonstrated any association between the exposure to RHD and number of episodes of illness in the subsequent 1 to 2 months.

The results of the study by Mead et al. may be summarized by noting that the average number of episodes of illness over the 13-month reporting period was 2.6 for respondents who had not been exposed to RHD virus, 2.2 for those classified as having a low level of exposure, and 2.3 for those classified as having a high level.

The study by Mead et al. concluded that, on the basis of the health survey and the lack of any serologic reaction of the respondents, there was considerable support to the view that RHD virus is not associated with infection or disease in humans. The results of the study have been submitted for publication in a scientific journal.

Reference 31 should refer to the Bureau of Resource Sciences (not Studies).

**C. Mead**
Convenor, Rabbit Calicivirus Human Health Study Group, Department of Health and Family Services, Canberra, Australia

### References

1. Smith AW, Skilling DE, Cherry N, Mead JH, Matson DO. Calicivirus emergence from ocean reservoirs: zoonotic and interspecies movements. Emerg Infect Dis 1998;4:13-20.
2. Mead C, Kaldor J, Canton M, Gamer G, Crerar S, Thomas S. Rabbit calicivirus and human health. Canberra, Australia: Department of Primary Industries and Energy, Australian Government (Released under the Official Information Act). Report of the Rabbit Calicivirus Human Health Study Group; 1996.

### Reply to Drs. Capucci, Lavazza, and Mead

**To the Editor:** We are aware of Capucci and Lavazza's excellent work. Indeed, one of the best characterized calicivirus genomes is that detected in rabbit hemorrhagic disease (RHD); however, the virus' infectivity, pathogenesis, modes of transmission, reservoirs, survival in nature, host of origin, virulence factors, number of neutralization serotypes, and multispecies infectivity are poorly characterized. Propagating this virus in vitro could provide insight for addressing questions relevant to caliciviruses that cannot be propagated in vitro.

We are unclear about the confusion regarding Norwalk virus and feline calicivirus (FCV). Both are caliciviruses. Norwalk virus is a human pathogen. FCV is in a different genus (1) that includes strains infecting humans (2). We know of no documented FCV infections in humans nor of detailed studies to search for such occurrences, although some evidence suggests the possibility (3).

Capucci and Lavazza's remaining questions address the etiology of RHD, diagnostic reagents, and possible human infection. They report nine laboratory workers as antibody negative but do not report test results on persons at high risk, such as rabbit farm workers, nor do they mention having positive control human or primate sera. Koch's postulates have been fulfilled for RHD: a parvovirus was isolated in vitro and was cell-passaged 15 times; at a second laboratory, the parvovirus was identified in materials causing RHD (4,5). In Europe the parvovirus etiology for RHD was deemed hypothetical but has not been refuted on a scientific basis. The calicivirus consistently identified in European materials has not been isolated in vitro, and Koch's postulates have not been fulfilled. Are the parvovirus-associated outbreaks of RHD in Mexico and China (4,5) and the calicivirus-associated RHD

outbreaks in Europe identical disease manifestations of two different viruses? Is RHD multifactorial requiring two or more agents? Is RHD caused by only a calicivirus or only a parvovirus? A calicivirus and a parvovirus can be isolated in vitro from the same fecal sample of a sick rabbit (N. Keefer, D.E. Skilling, A.W. Smith, unpub. data).

Our comments on RHD diagnostic assays referred to those used in Australia (6,7) to screen humans and experimentally infected animals to support legalizing the spread of RHD in Australia and New Zealand.

Public health protection requires prudent avoidance of pathogens associated with risk of adverse outcome, not necessarily proof of causation (8). In this context, human health risk for RHD goes largely unaddressed. The deliberate introduction of a new disease agent (RHD) known to cause death in mammals requires prudence rather than proof of human illness, especially when the scientific literature includes reports that the agent has induced antibody reactions in a wide range of mammalian and avian species (6).

Mead et al. (9) conclude, "No significant association between exposure to RCV and subsequent bouts of sickness could be demonstrated." Their recorded data do not support a statistically significant risk of illness because sample sizes in the monthly groups were too small for any meaningful interpretation. Mead et al. (9) state a "lack of any serologic reaction of the respondents," but a 50% cut-point was used for the competitive ELISA test, and some individual sera were repeated up to six times with percent inhibition reactions ranging from approximately 1% to 44% in one instance and 12% to 100% in another. Results were selected from these laboratory data and reported "lack of serologic reaction."

We derived our findings from data obtained under a freedom of information request. Mead et al. used the same data to support an opposite conclusion. Opposing conclusions "red flag" the quality of the study. In summary, the reporting of negative results of such a study cannot be used to support the important biologic, health, and political conclusion that humans are not at risk from infection with RHD.

We encourage a well-designed longitudinal study of persons at high risk of RHD exposure to answer conclusively whether RHD has infected humans. If "the rule" means that most humans exposed to RHD would become infected, we agree with Dr. Capucci "that infection is unlikely to be the rule," but transmission of equine morbillivirus, Rift Valley fever, and H5N1 influenza to humans is also "unlikely to be the rule" (10).

**Alvin W. Smith,* Neil J. Cherry,† and David O. Matson‡**

*Oregon State University, Corvallis, Oregon, USA; †Lincoln University, Christchurch, New Zealand; ‡Center for Pediatric Research, Norfolk, Virginia, USA

### References

1. Berke T, Golding B, Jiang X, Cubitt WD, Wolfaart M, Smith AW, et al. A phylogenetic analysis of the caliciviruses. J Med Virol 1997;52:419-24.
2. Smith AW, Berry ES, Skilling DE, Barlough JE, Poet SD, Berke T, et al. In vitro isolation and characterization of a calicivirus causing a vesicular disease of the hands and feet. Clin Infect Dis 1998;26:434-9.
3. Cubitt WD. Proceedings of the European Society of Veterinary Virology. Reading, United Kingdom: Reading University, Oct 1996.
4. Xu WY. Viral hemorrhagic disease in rabbits in the People's Republic of China: epidemiology and virus characterization. Rev Sci Tech, 1991;10:2393-408.
5. Gregg DA, House C, Meyer R, Berninger M. Viral haemorrhagic disease of rabbits in Mexico: epidemiology and viral characterization. Rev Sci Tech, 1991;10:2434-51.
6. Bureau of Resource Sciences, Australia. Rabbit calicivirus disease. Canberra, Australia: Australian Government Printing Office; 1996.
7. Collins BJ, White JR, Lenhaus C, Boyd V, Westbury HA. A competition ELISA for the detection of antibodies to Rabbit Hemorrhagic Disease Virus. Vet Microbiol 1995;43:85-96.
8. Brad-Hill A. The environment and disease: association or causation? Proceedings of the Royal Society of Medicine, 1965;58:295-300.
9. Mead C, Kaldor J, Canton M, Gamer G, Crerar S, Thomas S. Rabbit calicivirus and human health. Canberra, Australia: Department of Primary Industries and Energy, Australian Government (Released under the Official Information Act). Report of the Rabbit Calicivirus Human Health Study Group; 1996.
10. Vogel G. Sequence offers clues to deadly flu. Science News. Science 1998;279:324.

**To the Editor:** We are aware of Capucci and Lavazza's excellent work. Indeed, one of the best characterized calicivirus genomes is that detected in rabbit hemorrhagic disease (RHD); however, the virus' infectivity, pathogenesis, modes of transmission, reservoirs, survival in nature, host of origin, virulence factors, number of neutralization serotypes, and multispecies infectivity are poorly characterized. Propagating this virus in vitro could provide insight for addressing questions relevant to caliciviruses that cannot be propagated in vitro.

We are unclear about the confusion regarding Norwalk virus and feline calicivirus (FCV). Both are caliciviruses. Norwalk virus is a human pathogen. FCV is in a different genus (1) that includes strains infecting humans (2). We know of no documented FCV infections in humans nor of detailed studies to search for such occurrences, although some evidence suggests the possibility (3).

Capucci and Lavazza's remaining questions address the etiology of RHD, diagnostic reagents, and possible human infection. They report nine laboratory workers as antibody negative but do not report test results on persons at high risk, such as rabbit farm workers, nor do they mention having positive control human or primate sera. Koch's postulates have been fulfilled for RHD: a parvovirus was isolated in vitro and was cell-passaged 15 times; at a second laboratory, the parvovirus was identified in materials causing RHD (4,5). In Europe the parvovirus etiology for RHD was deemed hypothetical but has not been refuted on a scientific basis. The calicivirus consistently identified in European materials has not been isolated in vitro, and Koch's postulates have not been fulfilled. Are the parvovirus-associated outbreaks of RHD in Mexico and China (4,5) and the calicivirus-associated RHD outbreaks in Europe identical disease manifestations of two different viruses? Is RHD multifactorial requiring two or more agents? Is RHD caused by only a calicivirus or only a parvovirus? A calicivirus and a parvovirus can be isolated in vitro from the same fecal sample of a sick rabbit (N. Keefer, D.E. Skilling, A.W. Smith, unpub. data).

Our comments on RHD diagnostic assays referred to those used in Australia (6,7) to screen humans and experimentally infected animals to support legalizing the spread of RHD in Australia and New Zealand.

Public health protection requires prudent avoidance of pathogens associated with risk of adverse outcome, not necessarily proof of causation (8). In this context, human health risk for RHD goes largely unaddressed. The deliberate introduction of a new disease agent (RHD) known to cause death in mammals requires prudence rather than proof of human illness, especially when the scientific literature includes reports that the agent has induced antibody reactions in a wide range of mammalian and avian species (6).

Mead et al. (9) conclude, "No significant association between exposure to RCV and subsequent bouts of sickness could be demonstrated." Their recorded data do not support a statistically significant risk of illness because sample sizes in the monthly groups were too small for any meaningful interpretation. Mead et al. (9) state a "lack of any serologic reaction of the respondents," but a 50% cut-point was used for the competitive ELISA test, and some individual sera were repeated up to six times with percent inhibition reactions ranging from approximately 1% to 44% in one instance and 12% to 100% in another. Results were selected from these laboratory data and reported "lack of serologic reaction."

We derived our findings from data obtained under a freedom of information request. Mead et al. used the same data to support an opposite conclusion. Opposing conclusions "red flag" the quality of the study. In summary, the reporting of negative results of such a study cannot be used to support the important biologic, health, and political conclusion that humans are not at risk from infection with RHD.

We encourage a well-designed longitudinal study of persons at high risk of RHD exposure to answer conclusively whether RHD has infected humans. If "the rule" means that most humans exposed to RHD would become infected, we agree with Dr. Capucci "that infection is unlikely to be the rule," but transmission of equine morbillivirus, Rift Valley fever, and H5N1 influenza to humans is also "unlikely to be the rule" (10).
